# Supplementary material for: The Impact of Coilin Nonsynonymous SNP Variants E121K and V145I on Cell Growth and Cajal Body Formation: The First Characterization
Source: Genes (Basel). 2020 Aug 5;11(8):895. doi: 10.3390/genes11080895 (PMC7463897; doi:10.3390/genes11080895)
Supplement: Supplementary file 1 [file genes-11-00895-s001.zip › [Genes]Supplementary Figures proof (WEE) v2.0.pdf]

genes

MDPI

Supplementary figures for "The Impact of Coilin Nonsynonymous SNP Variants E121K and V145I on Cell Growth and Cajal Body Formation: The First Characterization"

A

rs116022828

1000_genomes ExAC

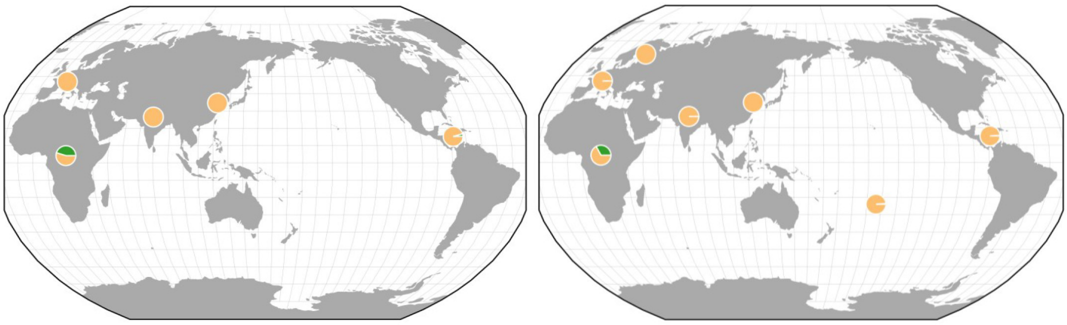

Population	Sample size	SNP frequency
Global	5008	0.013
**African**	**1322**	**0.045**
American	694	0.00
East Asian	1008	0.000
European	1006	0.000
South Asian	978	0.00

Population	Sample size	SNP frequency
Global	120954	0.00291
**African**	**10172**	**0.0330**
American	11578	0.0010
Asian	25164	0.0000
European	73134	0.0000
Other	906	0.00

B

rs61731978

1000_genomes ExAC

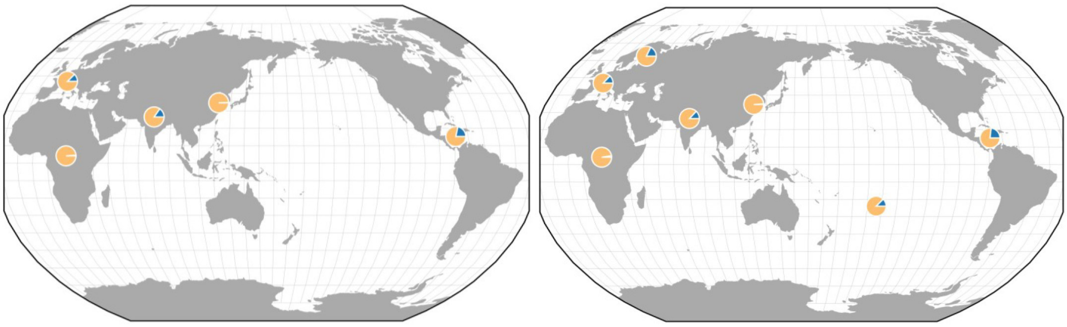

Population	Sample size	SNP frequency
Global	5008	0.089
African	1322	0.010
**American**	**694**	**0.22**
East Asian	1008	0.005
European	1006	0.125
South Asian	978	0.15

Population	Sample size	SNP frequency
Global	120954	0.12417
African	10172	0.0255
**American**	**11578**	**0.2324**
Asian	25164	0.0882
European	73134	0.1334
Other	906	0.13

Figure S1: Global distributions of *COIL* SNPs, (**a**) rs116022828 (E121K) and (**b**) rs61731978 (V145I), analyzed using multiple datasets from various studies in the Single Nucleotide Polymorphism Database (dbSNP; https://www.ncbi.nlm.nih.gov/snp/). Summary of two studies is given: 1000 Genome Project Consortium (1000_Genomes) and Exome Aggregation Consortium (ExAC). The population with the highest SNP frequency in each dataset is highlighted in bold. World maps were generated and adapted from the Geography of Genetic Variants browser (https://popgen.uchicago.edu/ggv) [54].

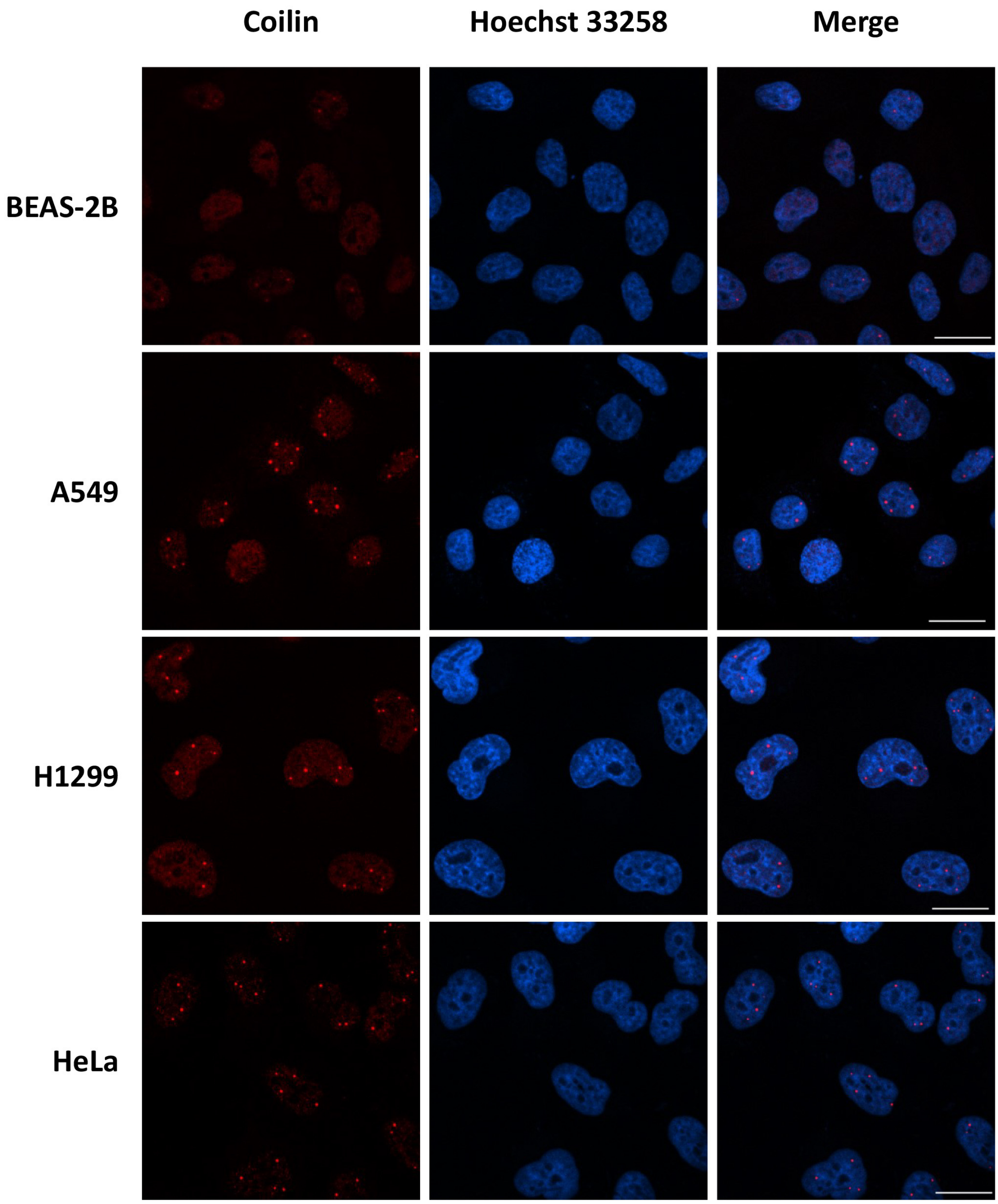

Figure S2: Immunofluorescence analysis of CB formation in a normal human bronchial epithelial cell line (BEAS-2B), two lung cancer cell lines (A549 and H1299), and a cervical cancer cell line (HeLa) probed with coilin antibody (sc-55594, Santa Cruz Biotechnology); DNA was stained with Hoechst 33258. Scale bar = 20 μm.

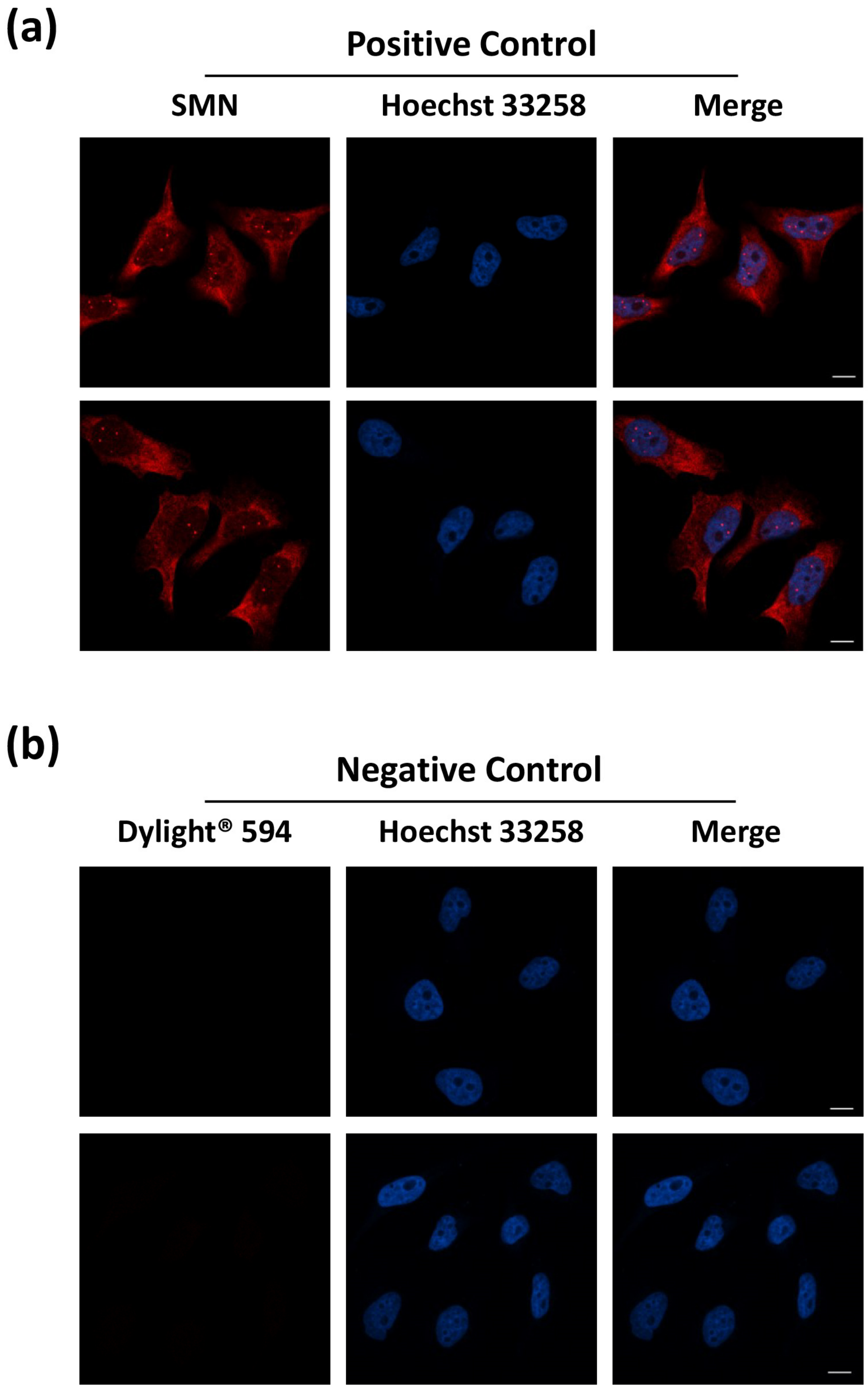

Figure S3: Specificity of SMN antibody in immunofluorescence analysis. (**a**) Positive control: parental HeLa cells were probed with SMN antibody (#12976, Cell Signaling Technology) followed by Dylight® 594 (35510, Thermo Fisher Scientific) staining. (**b**) Negative control: parental Hela cells were stained directly with Dylight® 594; scale bar = 10 μm.

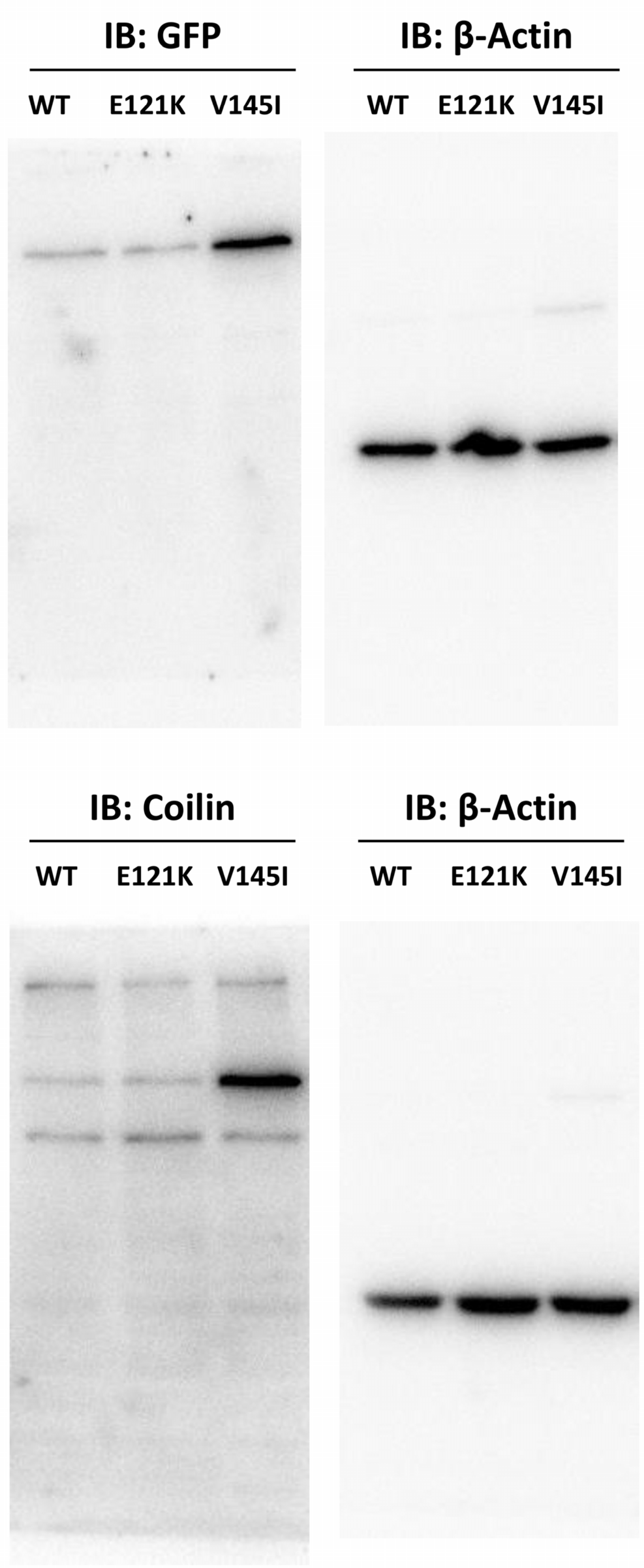

Figure S4: Immunoblotting of coilin WT and SNP variant (E121K and V145I) cells probed with GFP (sc-9996, Santa Cruz Biotechnology), coilin (sc-55594, Santa Cruz Biotechnology), or β-actin (A5441, Sigma-Aldrich) antibody, display in full blots.

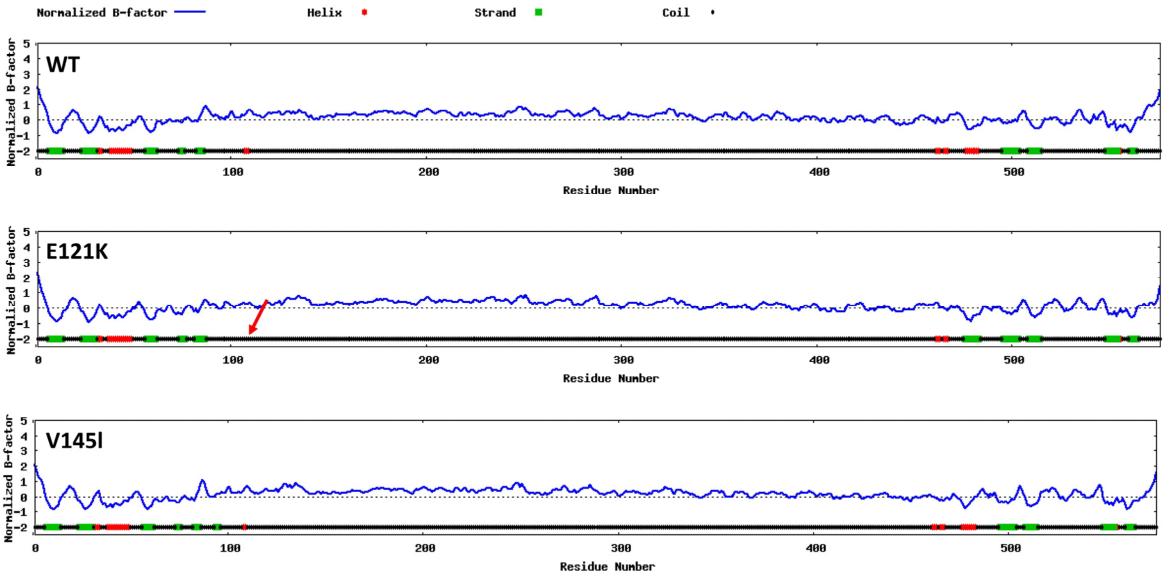

Figure S5: Predicted normalized B-factors generated by I-TASSER server (https://zhanglab.ccmb.med.umich.edu/I-TASSER) based on the amino acid sequences of the coilin WT and SNP variants [28]. B-factor is a value to indicate the extent of the inherent thermal mobility of residues in proteins. Negative value means the residue is relatively more stable in the structure. Red arrow indicates the position where the predicted helix structure is missing in the E121K mutant.

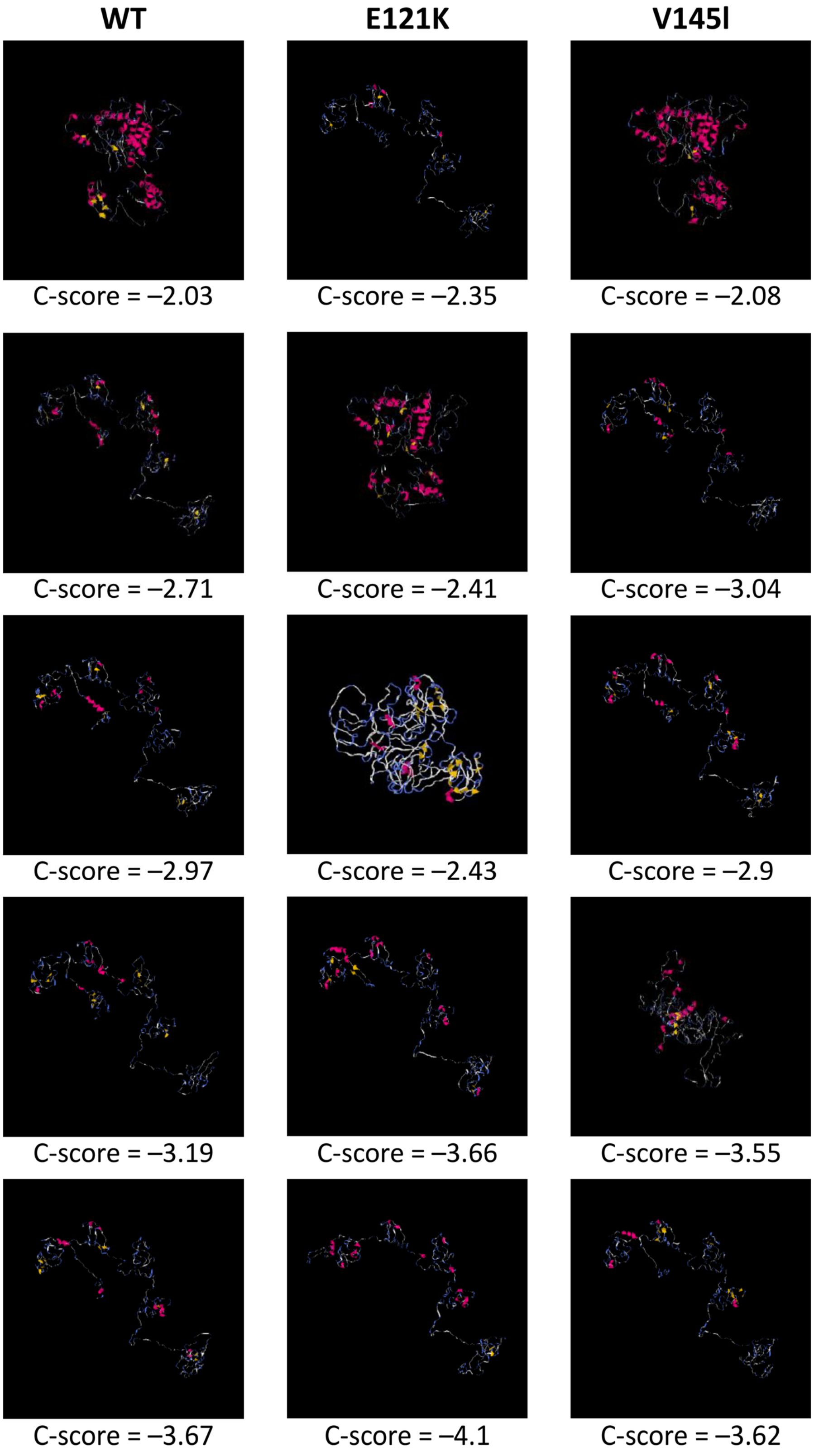

Figure S6: Top five models predicted by I-TASSER server (https://zhanglab.ccmb.med.umich.edu/ I-TASSER) based on the amino acid sequences of the coilin WT and SNP variants (E121K and V145I). C-score is typically in the range of –5 to +2, and a C-score > –1.5 indicates a model of correct global topology [28].
